# Photovoltaic and Photomultiplication Dual‐Mode Near‐Infrared Organic Detectors with Large Dynamic Range for Intensive and Faint Light Sensing

**DOI:** 10.1002/advs.202506499

**Published:** 2025-05-30

**Authors:** Xin Hu, Ning Li, Mingyang Ren, Yifan Ji, Hongmei Guo, Qian Chen, Xiubao Sui

**Affiliations:** ^1^ School of Electronic and Optical Engineering Nanjing University of Science and Technology 200 Xiaolingwei Street Nanjing 210094 China; ^2^ School of Computer and Electronic Information Nanjing Normal University Nanjing 210023 China

**Keywords:** large dynamic range, organic photodetectors, photovoltaic/photomultiplication dual‐mode, near‐infrared

## Abstract

Conventional detectors usually operate in one detection mode, namely photovoltaic mode, photoconductive mode, or photomultiplication mode, etc., which may have limitations such as insufficient sensitivity and limited dynamic range for applications. In this study, a photovoltaic/photomultiplication dual‐mode organic photodetector is developed by integrating two different organic bulk heterojunctions (BHJs), one introduces the photovoltaic effect, and the other triggers photomultiplication charge generation in the stacked device at different voltage polarities. The combination of the two operation modes largely extends the dynamic range of detectors, even superior to that of a commercial silicon detector. The ultra‐low noise level of the photovoltaic mode enables it to detect faint light with exceptional sensitivity, while the photomultiplication mode can handle a significantly large saturation photocurrent, allowing for the precise detection of high‐intensity signals. A dynamic range of 225 dB is achieved by precisely controlling the operation conditions of the dual‐mode detector, which surpasses the dynamic range of 140 dB of a commercial silicon detector. The detector can, as a result, precisely detect light signals (800 nm) in the range of 10^−10^–10 W cm^−2^. This study demonstrates the versatility of organic semiconductors in designing multi‐functional and high‐performing photodetectors for practical applications.

## Graphical abstract

A photovoltaic/photomultiplication dual‐mode organic photodetector is developed by integrating two different organic bulk‐heterojunctions, one introduces the photovoltaic effect, and the other triggers photomultiplication charge generation in the stacked device at different voltage polarities. A dynamic range of 225 dB is achieved by precisely controlling the operation conditions of the dual‐mode detector, surpassing the dynamic range of a commercial silicon detector.

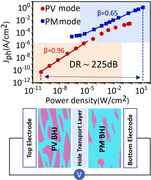

## 1 Introduction

Light‐sensing techniques underpin modern technology. Specifically, near‐infrared (NIR, 0.8–1.5 µm) photodetectors play a crucial role in current science and technology in terms of industry, astronomy, medicine, defense, and other fields.^[^ ^1^, ^2^, ^3^, ^4^, ^5^ ^]^ Inorganic semiconductor materials have been used to make NIR detectors for a long time. For example, silicon detectors have been made to detect NIR signals due to their low noise level and low cost.^[^ ^6^, ^7^, ^8^ ^]^ The silicon detectors are usually limited to 1.1 µm since silicon has a bandgap of 1.12 eV. Indium Gallium Arsenide is developed to extend the photoresponse spectrum, showing a typical response spectrum of up to 1.7 µm.^[^ ^9^ ^]^ These detectors are mature and ubiquitous in modern cameras. However, these inorganic material‐based detectors are usually rigid and fragile, hindering their applications in flexible and wearable electronics. In recent years, organic photoelectric materials have become an attractive alternative for NIR light sensing due to their advantages of large absorption coefficient, lightweight, flexibility, and tunable optical properties.^[^ ^10^, ^11^, ^12^ ^]^ Efficient light detection utilizing organic semiconductors has been realized by rational design of the material compositions and detector structures.^[^ ^13^, ^14^, ^15^ ^]^ Meanwhile, by controlling the morphology of the bulk heterojunction (BHJ), the performance parameters of organic photodetectors can be significantly optimized.^[^ ^16^, ^17^ ^]^ Such morphological engineering strategies provide new insights for balancing device performance with processing feasibility.

Metrics of photodetectors include quantum efficiency, dynamic range, noise level, response speed, etc. Specifically, the dynamic range of a detector measures the range of detectable light intensity, from intense to faint light signals, which represents the capability to differentiate between different targets. The dynamic range of a photodetector is usually dominated by material properties and device structures.^[^ ^18^, ^19^, ^20^ ^]^ For example, materials with low defect density (such as perovskite^[^ ^21^, ^22^ ^]^) are favorable for making detectors with reduced noise and thus improved dynamic range. On the other hand, properly designing the device structure, such as adopting multi‐layer structures, can enhance carrier collection efficiency, which also improves dynamic range.^[^ ^23^, ^24^ ^]^ Such methods, to a certain extent, can improve the dynamic range of a detector, but they impose high demands regarding material purity and cost. Backend signal processing uses advanced algorithms for signal enhancement and noise suppression, which can also improve the dynamic range of a detector.^[^ ^25^ ^]^ However, the introduction of complex algorithms may increase processing latency, making real‐time applications more challenging.

To further address the challenges in achieving a wide dynamic range, it is essential to engineer the device structure and introduce innovative operation mechanisms. Researchers have ingeniously designed an organic photodetector capable of operating in two modes, i.e., photovoltaic (PV) and photomultiplication (PM) modes. The two operating modes can be swapped by an external bias.^[^ ^15^, ^26^ ^]^ By switching between the two operating modes, the performance of the detector can be significantly extended. This provides valuable inspiration for the design of the detector presented in this work. Herein, a PV detector and a PM detector are integrated into a back‐to‐back structure to realize the two operating modes under different bias polarities. Different from previous designs, we have employed two distinct light‐absorbing layers, thereby enabling more flexible tuning of the detector's response spectrum. It is found that the combination of PV and PM effects in organic material‐based NIR detectors can dramatically enlarge the dynamic range by exploiting the advantages of both operation modes: the PV mode with a low noise level for faint light detection and the PM mode for intensive signal sensing due to its large saturation photocurrent. A PV/PM dual‐mode photodetector with a large dynamic range of 225 dB is demonstrated by exploiting two organic semiconducting bulk‐heterojunctions integrated into a stacked device structure. The PV and PM BHJs are integrated in a compact configuration, operating individually depending on the polarity of the external bias. The responsivity (R) and detectivity (D*) of the dual‐mode detector reach 0.3 A W^−1^ and 2.5 × 10^11^ Jones when operating in the PV mode, and 2.38 A W^−1^ and 1.59 × 10^9^ Jones in the PM mode, under 800 nm light. The detector showcases a solid detection range of 10^−10^ to 10 W cm^−2^ with a dynamic range of 225 dB in the NIR wavelength band, fully combining the benefits of the PV and PM modes. The performance of the dual‐mode organic detector exceeds that of commercial silicon‐based detectors in terms of responsivity and dynamic range, since a typical silicon detector exhibits a responsivity of 0.5 A W^−1^ and a dynamic range of 140 dB at 800 nm. The physical mechanisms and optimization methods to improve the performance of the dual‐mode detector are also explored. The high dynamic range imaging using the dual‐mode detector is successfully demonstrated in the NIR range, indicating its potential for practical applications.

## 2 Results

### 2.1 Design Concepts of the PV/PM Dual‐Mode Detector

**Figure 1:**
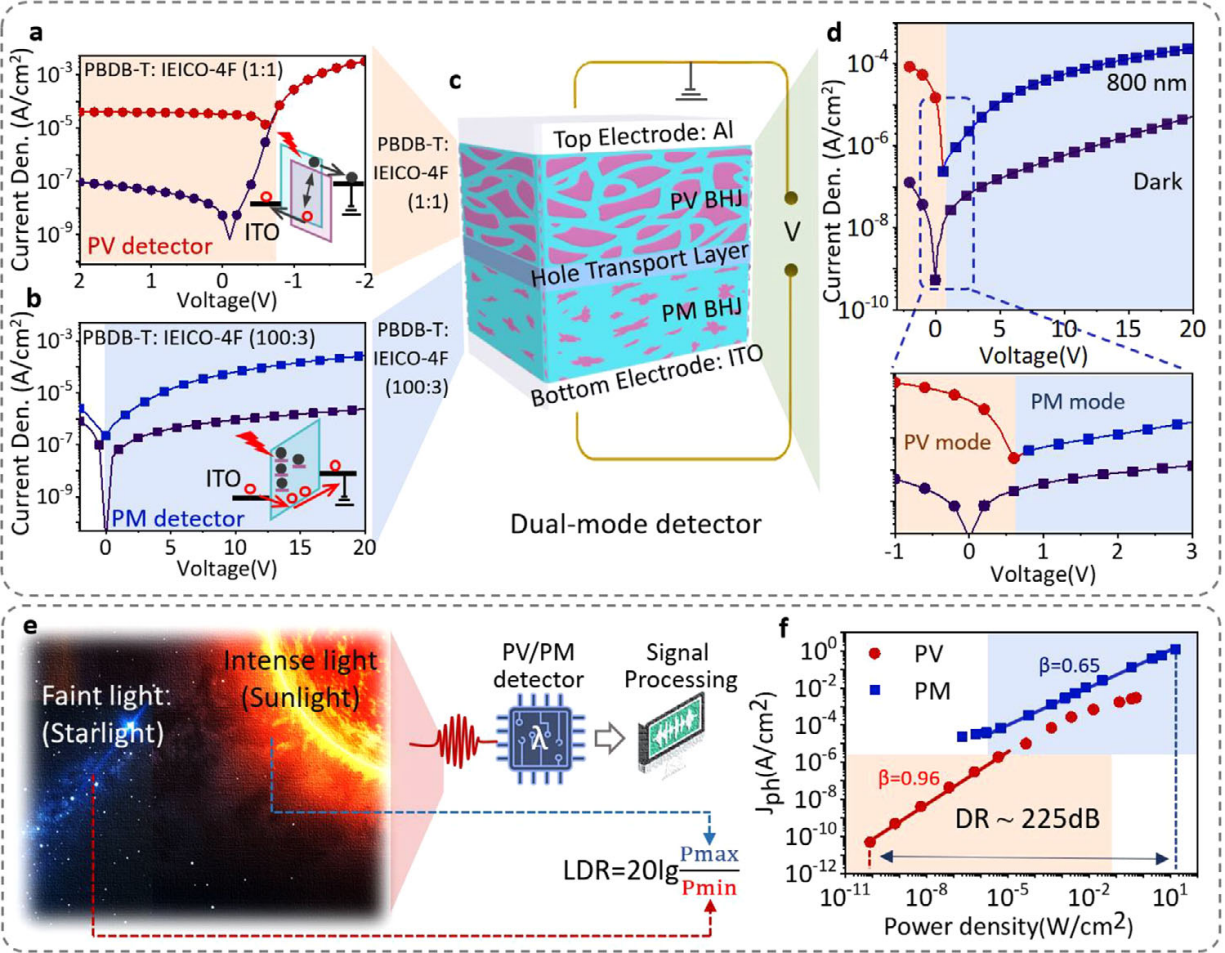
Design concept of the high dynamic range NIR PV/PM dual‐mode detector. The current density‐voltage characteristics of a) a PV detector, and b) a PM detector in the dark and under 800 nm light. The figures in the inset schematically show the working mechanism of the detectors. c) The schematic structure of the dual‐mode detector, comprising a bottom electrode, a multiplier layer (PM BHJ), a hole transport layer, a photovoltaic layer (PV BHJ), and a top electrode. d) The current density‐voltage curves of the PV/PM dual‐mode infrared detector in dark and under 800 nm light. e) Schematics to show the dual‐mode detector in detecting faint and intense optical signals, e.g., starlight and sunlight. A photodetector with a large dynamic range is required. f) The relationship between incident light power density and photocurrent density of the dual‐mode detector. The dynamic range is estimated to be 225 dB. The incident light intensity of measurements in parts a) and b) is set at 0.068 mW cm^−^^2^. The light intensity in part d) is set at 0.24 mW cm^−2^.

Organic BHJs have been actively investigated to tune the PV/PM effect in a photodetector by modulating the donor‐acceptor ratio.^[^ ^27^, ^28^ ^]^ PBDB‐T and IEICO‐4F are donor‐acceptor materials used in the active BHJs in this work. The absorbance, full names, and molecular structures of the two materials are provided in Figure S1 (Supporting Information). We selected the polymer donor material PBDB‐T and the non‐fullerene acceptor material IEICO‐4F to construct the BHJ, primarily based on the following two considerations. First, in terms of spectral characteristics, PBDB‐T exhibits strong absorption in the 500–650 nm visible range, while IEICO‐4F shows characteristic absorption in the 700–900 nm near‐infrared region, demonstrating excellent spectral complementarity and enabling broad‐spectrum response, as illustrated in Figure S1a (Supporting Information). Second, the dispersed IEICO‐4F molecules in the BHJ can trap photogenerated electrons to enable photomultiplication. These trapped electrons near the Al electrode generate a strong Coulomb potential field, inducing interfacial band bending and facilitating hole tunneling injection, thereby achieving remarkable photomultiplication. These features underpin the development of the dual‐functional PV/PM device. **Figure** **1** schematically shows the design roles of the PV/PM dual‐mode device. The dual‐mode detector consists of two diode‐like parts stacked in a back‐to‐back structure with a layer configuration of ITO/PBDB‐T: IEICO‐4F (100:3, w/w)(450 nm)/ PEDOT: PSS(100 nm)/PBDB‐T: IEICO‐4F (1:1, w/w)(250 nm)/LiF(2 nm)/Al(100 nm), as shown in Figure 1c. The back‐to‐back integration approach is not particularly challenging for solution‐processed semiconductor devices.^[^ ^14^, ^29^ ^]^ However, at present, the integration of PV and PM detectors to combine the two working modes remains unexplored. In general, the PEDOT: PSS layer connecting the two parts not only serves as a hole transport interlayer but also protects the underlying active BHJ layer from the erosion of the solvent of the upper layer during the spin‐coating process when fabricating the device. The energy diagrams for all materials used in this work are illustrated in Figure S2 (Supporting Information).

Before investigating the dual‐mode detector, the response behaviors of the two control detectors made with two distinct BHJs with PBDB‐T/IEICO‐4F weight ratios of 1:1 and 100:3 are studied. The PV detector has a structure of ITO/PEDOT: PSS/PBDB‐T: IEICO‐4F (1:1)/LiF/Al, and the PM detector has a structure of ITO/PBDB‐T: IEICO‐4F (100:3)/PEDOT: PSS/LiF/Al. The current density‐voltage characteristics of the PV and PM control detectors are illustrated in Figure 1a,b. The PV detector exhibits a typical photovoltaic effect, featuring an open circuit voltage of ≈0.75 V due to its built‐in potential induced by the donor and acceptor interface. The non‐zero built‐in potential enables the detector to generate photocurrent without external bias. Nevertheless, photogenerated electrons and holes can be more efficiently collected by applying a reverse bias. There is a clear saturation feature in the detector's photocurrent as external bias increases, indicating the detector has limited current transport capacity. As a comparison, the PM detector shows an increased photocurrent, which benefits from a charge injection mechanism. The distribution of the low concentration IEICO‐4F within the binary blended PBDB‐T: IEICO‐4F (100:3) active layer creates IEICO‐4F‐induced electron traps, which lowers the hole injection barrier between the ITO electrode and the BHJ layer. This electron accumulation‐induced band bending gives rise to the photomultiplication effect once more than one charge is injected upon one‐photon absorption. The PM effect exhibits a continuous increase in photocurrent as the voltage rises, with no saturation observed even at high external bias levels. As expected, there is almost no photocurrent at 0 bias, which distinguishes the PV and PM modes. Meanwhile, it is observed that the dark current in the PM detector is higher than that in the PV detector at their respective optimal operating voltages. From the lower dark current, it can be inferred that the PV effect exhibits a lower noise level since the dark current noise dominates the total noise of the device.

As demonstrated in Figure 1a,b, the PV‐type organic detector has a lower dark current, which is favorable for detecting weaker light signals. The PM‐type organic detector can support higher photocurrents, making it adept at detecting stronger light signals. Therefore, the two types can complement each other to achieve the detection of light signals over a large dynamic range. In this work, a back‐to‐back structure comprising a PV detector and a PM detector is designed, as illustrated in Figure 1c. The two detectors are bridged by a hole transport layer, allowing the operational modes to be selectively adjusted by simply reversing the polarity of the applied external bias. The current density‐voltage curves of the dual‐mode detector in dark and light conditions as a function of bias voltages are illustrated in Figure 1d. The current in the dark and under light combines the characteristics of the two control detectors. Under reverse biases, the dual‐mode detector shows a PV effect, with a distinguishable open circuit voltage of ≈0.6 V. The detector exhibits the PM effect at positive biases, demonstrating a continuously increasing photocurrent with increasing bias. The current‐voltage characteristics of the integrated detector indicate the operation of dual modes. It is observable that the PV mode exhibits low dark current, indicating that a low noise level can be attained, which is favorable for weak signal detection. Meanwhile, the PM mode demonstrates a high photocurrent capacity without saturation even at high bias, indicating that the PM mode is conducive to detecting strong light signals.

As shown in Figure 1e, detectors frequently encounter the challenge of detecting faint signals such as starlight from a distant galaxy, and sudden intense signals such as sunlight, explosions, and lightning. These situations impose higher demands for detectors with large dynamic ranges. The linear dynamic range (LDR) is described in the following equation,^[^ ^21^ ^]^

(1) $$\mathrm{LDR}=20\mathrm{lg}\frac{\mathrm{Pmax}}{\mathrm{Pmin}}$$

where P_max_ and P_min_ represent the maximum and minimum values of detectable light intensity in a linear range.

The disparity between the PV and PM modes, as demonstrated in Figure 1d has prompted the development of dual‐mode detectors capable of achieving a high dynamic range by exploiting the low noise level in PV mode and high photocurrent capacity in the PM mode, thereby avoiding the requirement for complex optical designs and algorithmic corrections. The relationship between incident light power density and photocurrent density of the dual‐mode detector is studied, and the results are depicted in Figure 1f. Considering the low noise level, the PV mode is employed for weak signal detection in the dynamic range test. As shown in Figure 1f, the PV mode enables the detection of incident light power densities as low as 10^−10^ W cm^−2^. When the light intensity exceeds 10^−5^ W cm^−2^, the response deviates from a linear dependence on the light intensity, demonstrating a dynamic range of only ≈100 dB for the PV mode. Under these circumstances, the PM mode, which boasts a significantly higher saturation photocurrent, can be effectively utilized to enhance the detector's capacity for handling intense light signals. A forward bias of 20 V is applied to trigger the PM mode. As presented in Figure 1f, the highest measurable incident light power density is 10 W cm^−2^ for the PM mode. However, due to the relatively higher dark noise level in the PM mode, the lower threshold of photoresponse corresponds to a light intensity of 10^−5^ W cm^−2^. The PM mode demonstrates an impressive dynamic range of 129 dB, effectively broadening the dynamic range of the PV mode. This enhancement results in a combined dynamic range of 225 dB for the dual‐mode detector, offering superior performance across a wide spectrum of light intensities from 10^−10^ to 10 W cm^−2^. From the current density‐power density relationship in a double‐logarithmic scale, the slope values (β) for the device in PV mode and PM mode are estimated to be ≈0.96 and ≈0.65, respectively. It's noteworthy that the detector demonstrates a sublinear response to irradiance, which emulates the human eye's response, a feature that holds great promise for future applications.^[^ ^30^ ^]^ This represents the highest reported dynamic range for organic infrared detection, surpassing that of traditional silicon‐based photodetectors. Figure S3 (Supporting Information) shows the dynamic range data measured from a commercial silicon detector. The results indicate that the noise current of this silicon detector is ≈1 nA, and the saturation photocurrent is ≈10 mA. The dynamic range for this silicon detector is estimated to be ≈140 dB. The dynamic range of this silicon detector is lower than that of our dual‐mode detector. The photocurrent of our detector outperforms that of the commercial silicon detector under both low and high light intensity conditions.

It should be noted that the photocurrent density in PM mode displays a sub‐linear relationship with incident light power density due to the introduction of traps in the PM BHJ, potentially impeding the transfer of photo‐induced charge carriers. However, by taking advantage of the larger saturated photocurrent in the PM mode, we can still enhance the dynamic range of the detector by easily switching between the two modes, thereby more effectively detecting light signals over a broader power range.

### 2.2 Device Physics and Performance of the Dual‐Mode Detector

**Figure 2:**
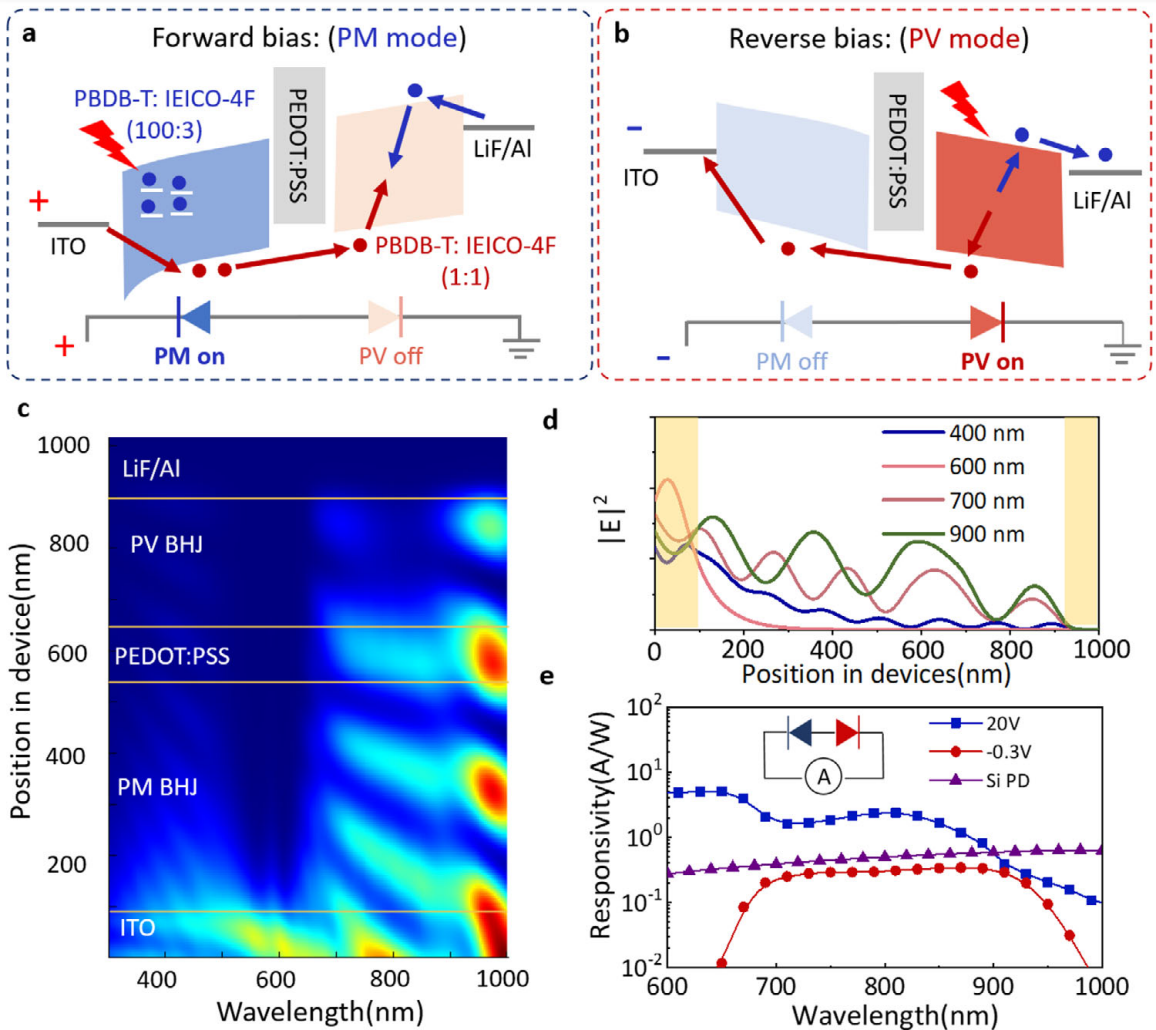
Working mechanism and light response characteristics of the dual‐mode detector. The charge transport dynamics of the dual‐mode detector at a) forward bias, and b) reverse bias. The PM mode is on at forward bias while the PV mode is on at reverse bias. c,d) Simulation of light field distribution as a function of wavelength and position in the dual‐mode detector. e) The responsivity results of the dual‐mode detector operating in PM (20 V) and PV (−0.3 V) modes. The results are compared to the responsivity of a silicon detector (Thorlabs DET36A2).

The large dynamic range of the dual‐mode detector is enabled by integrating the PV and PM BHJs into a single device with a back‐to‐back structure. This structure allows the transition of the two modes by switching the polarity of the external bias. The working mechanism of the dual‐mode detector is illustrated in **Figure** **2**a,b. The PV BHJ (PBDB‐T and IEICO‐4F with a weight ratio of 1:1) and PM BHJ (PBDB‐T and IEICO‐4F with a weight ratio of 100:3^[^ ^31^ ^]^) are connected by a p‐type layer (PEDOT: PSS). The external bias is applied to the ITO electrode, with the aluminum electrode grounded.

In the forward bias condition, the PM BHJ operates in reverse bias while the PV BHJ operates in forward bias, as illustrated in Figure 2a. Under light illumination, charges are generated in both the PM BHJ and PV BHJ. However, the forward bias on the PV BHJ will force the photogenerated charges in the PV BHJ to recombine, which does not contribute to the photocurrent. On the contrary, the photogenerated charges in the reverse‐bias PM BHJ contribute to the photocurrent. The photogenerated electrons within the PM BHJ are captured by isolated traps formed by IEICO‐4F, a phenomenon attributed to the minimal presence of IEICO‐4F in the BHJ matrix. These trapped electrons at the ITO/PM BHJ interface induce a band‐bending effect, which effectively reduces the hole injection barrier between ITO and the PM BHJ. This reduction facilitates a substantial hole injection through a hole tunneling injection mechanism, thereby enhancing the overall photocurrent response. The injected holes transport through the intermediate layer PEDOT: PSS, and recombine with the electrons injected from the aluminum side in the PV BHJ. The PV BHJ serves as a low‐resistance channel for charge transport in this condition. The band‐bending induced hole injection in the PM BHJ is very effective, giving rise to the photo multiplication phenomenon, where the absorption of a single photon results in the transport of multiple charge carriers.

In the reverse bias condition, the PM BHJ enters a forward conductive state, serving as a hole transport layer for the charges generated in the PV BHJ, as demonstrated in Figure 2b. Electrons generated in the PV BHJ under light migrate toward the aluminum electrode, and holes transport through PEDOT: PSS and PM BHJ layers, being collected by external circuits, forming the current flow. In the PV mode of the detector, the photogenerated holes can pass through the PEDOT:PSS and the PM active layer under external bias, because the PEDOT:PSS layer is the hole transporting layer, and the PM active layer is also p‐type. This is because the PBDB‐T material dominates in the PM active layer. The energy diagram of the PBDB‐T indicates that its highest occupied molecular orbital level (−5.53 eV) is pretty aligned with the work function of the PEDOT:PSS (−5.1 eV) layer. There is an energy barrier of only 0.4 eV. Given the open‐circuit voltage of 0.75 V in the PV detector as shown in Figure 1a, the built‐in potential is already large enough to help photogenerated holes overcome the energy barrier between PEDOT:PSS and PBDB‐T. Though light‐induced charge generation occurs in the PM BHJ, it does not contribute to the photocurrent, because the external bias compels these charges to recombine within the PM BHJ before being collected by the external circuits.

It should be noted that the PM layer may act as an optical filtering layer when the dual‐mode detector operates in the PV mode, since the light is illuminated from the ITO side, and photons to be detected need to pass through the PM BHJ layer. The profile graph in Figure 2c depicts the distribution of light intensity as a function of wavelength and position in the dual‐mode detector. It is observed that incident light with wavelengths of <650 nm gradually weakens after traversing the PM BHJ, allowing photons with wavelengths of >650 nm to reach the PV layer. The wavelength‐dependent light intensity profiles as a function of position in the dual‐mode detector, as provided in Figure 2d confirm the analysis of the results in Figure 2c. The light distribution in the detector can certainly affect the spectral photoresponse in the PV mode of the detector.

The responsivity of the dual‐mode photodetector operating in the PM mode (20 V) and PV mode (−0.3 V) is presented in Figure 2e. The results are compared to those of a silicon photodetector. It is clear that the PM mode exhibits a much higher responsivity due to the photomultiplication effect. The corresponding external quantum efficiency (EQE) results for the PM and PV modes are presented in Figure S4 (Supporting Information). The responsivity in the PM mode at 800 nm is 2.38 A W^−1^, corresponding to an external quantum efficiency of 370% calculated from the *EQE*  =  *R* · *h*ν relationship, where *h*ν is the photon energy with the unit of eV. The responsivity in the PV mode is comparable to that of a silicon photodetector, but the response profile shows a wavelength‐selective feature, which is due to the internal filtering effect as previously discussed. Visible‐blind NIR detectors are promising in bio‐medical related applications as in medical applications, highly sensitive infrared signal detection and imaging, which require shielding from visible light interference, have found widespread uses, particularly in tumor biomarkers and drug therapy.^[^ ^32^ ^]^ Research has identified the spectral range between 650 and 900 nm as the optimal optical window for biological detection and imaging, which greatly avoids absorption by biological tissues (hemoglobin and water) and aligns with the designed detection range of the detector. The stacked structure design and internal filtering effect in our detector may provide a solution for these application settings. The EQE values of the PV‐mode detector and silicon detector are 46.5% and 77% at 800 nm, respectively, much lower than that of the PM‐mode detector (370%).

The EQE results of the PV and PM control detectors as a function of wavelength are shown in Figure S5 (Supporting Information). It can be observed that both devices have broadband photoresponse covering the wavelength range from 350 to 1000 nm. The EQE of the PV device saturates in the −0.5–−2 V bias range, and the EQE values are <100% over the whole spectrum. The PM control device, on the contrary, exhibits EQE values over 100% in the spectral range of 350–850 nm at optimal bias voltages, without any saturation phenomenon as bias increases. These results correspond well with the data in Figure 1a,b. In contrast to the relatively flat photoresponse observed in the PV detector, the PM detector exhibits a lower EQE in the NIR band compared to the visible band. This discrepancy is primarily attributed to the low concentration of IEICO‐4F, which predominantly contributes to NIR absorption in the BHJ.

**Figure 3:**
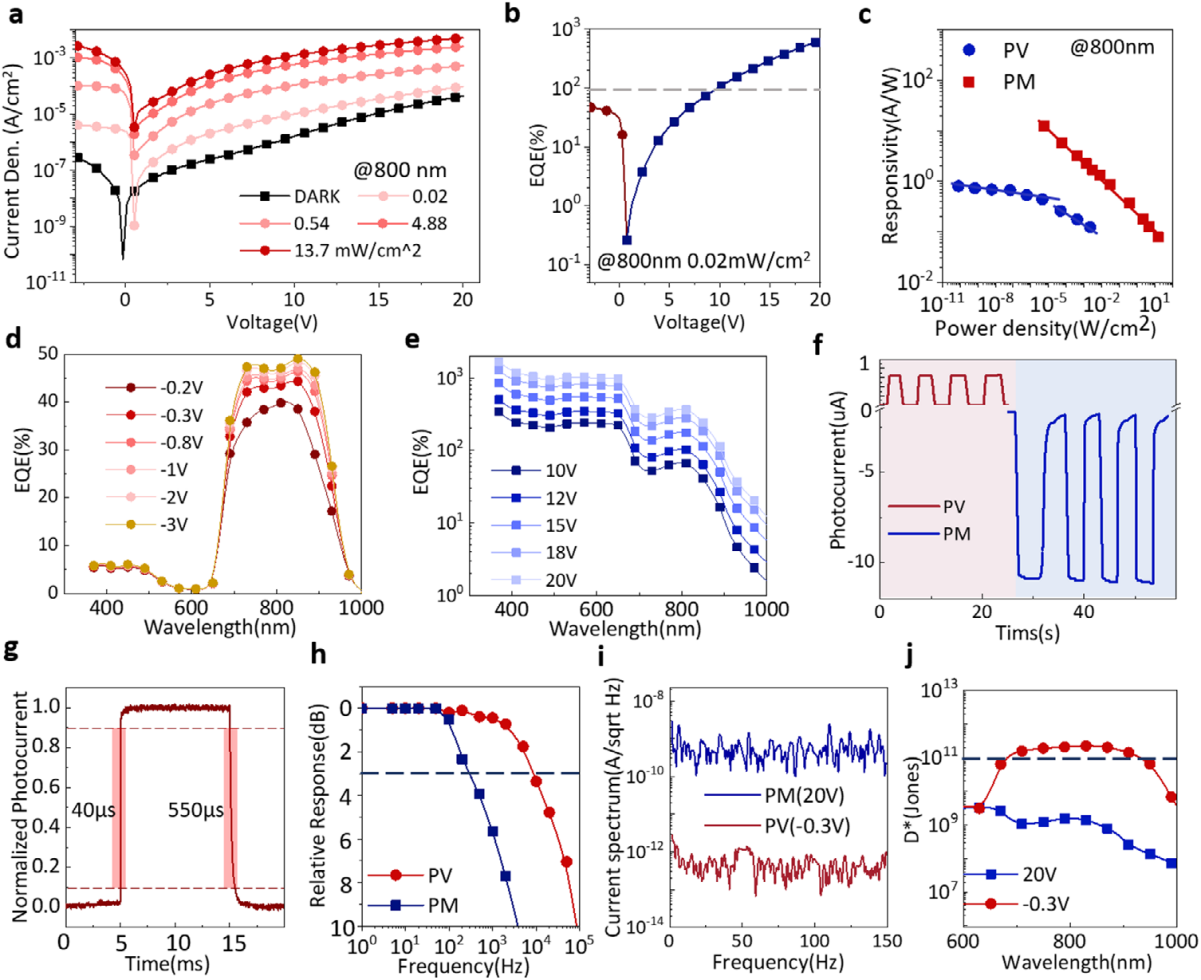
Optoelectronic performances of the dual‐mode detector. a) Current density–voltage curves at different light intensities. b) An example of EQE curve at different voltages under 800 nm illumination (0.02 mW cm^−2^). c) Relation between responsivity and incident light intensity. d) PV type, and e) PM type EQE in the visible‐near‐infrared range. f) The transient photocurrent measured at two typical voltages in PV (−0.3 V) and PM (20 V) modes, under 800 nm light (0.02 mW cm^−2^). g) Response time estimation in the PV mode. h) 3 dB cut‐off frequency estimation for PM and PV modes. i) Spectrum noise current measured at PM and PV modes. j) Specific detectivity (D^*^) of the dual‐mode detector operating in PM and PV modes.

The performances of the dual‐mode detector are further investigated. **Figure** **3**a shows the current response of the dual‐mode detector to NIR light (800 nm) as a function of bias. The NIR light is varied from 0.02 to 13.7 mW cm^−2^. The dual‐mode detector operates in PV mode at reverse biases, with a distinguishable open‐circuit voltage of ≈0.6 V. The photocurrent reaches a high level even at relatively low biases, demonstrating the effect of the internal built‐in potential. The detector operates in PM mode with forward biases, with less saturation in the photocurrent as compared to that from the PV mode. An increasing photocurrent generation occurs in the PM mode by applying higher voltages, suggesting that deeper electron traps formed by IEICO‐4F can capture a substantial number of NIR‐induced electrons and trigger an efficient hole tunneling injection, ultimately leading to considerable device multiplication. The current density‐voltage curves of the dual‐mode detector match those of single BHJ detectors (Figure 1a,b). Therefore, the dual‐mode detector can combine the advantages of the different operation modes, paving the way for multi‐functional application settings.

Figure 3b is an example of EQE as a function of bias for the dual‐mode detector. The EQE in the PV mode quickly gets saturated, and the final EQE is below 100%. On the contrary, the EQE in the PM mode increases with bias and surpasses the 100% benchmark. The responsivity of the dual‐mode detector as a function of incident light intensity is calculated from Figure 1f, and results are presented in Figure 3c. The results are recorded at 20 V for PM mode and −0.3 V for PV mode. It can be observed from Figure 1f that the photocurrent of the device changes more significantly with light intensity in the PV mode. The slope values in both modes are <1, indicating the change in the responsivity of the detector under different light intensities. The results in Figure 3c confirm this estimation. The primary reason for this phenomenon is the presence of trap states within the detector. When the light intensity increases, the number of carriers inside the detector also increases. Under the influence of defect states, the recombination rate of photogenerated electrons and holes increases, thereby reducing the responsivity of the detector.^[^ ^10^ ^]^

Figure 3d,e displays the EQE spectra for the PV and PM modes of the dual‐mode detector. Again, the saturation in the photoresponse is more evident in the PV mode than in the PM mode as bias increases. The enhancement of EQE with increasing bias in the PM mode is attributed to strengthened hole injection and improved hole transport in the active layer at higher biases. At a bias of −0.3 V, the EQE almost attains the highest value of the PV mode. At a bias of 20 V, the EQE reaches 1000% in the visible range (500 nm) and 370% in the NIR region (800 nm).

The EQE spectra exhibit a broadband response in the PM mode, contrasting with the NIR wavelength selectivity observed in the PV mode. This observation aligns well with our previous discussions. The internal optical filtering effect caused by the PM BHJ results in the distinct EQE spectral shapes of the detector in the two modes. By simply adjusting the applied voltage of the detector, rapid switching between detection modes can be realized. The photocurrent versus time curves in Figure 3f present different photocurrent directions in the PV (−0.3 V) and PM (20 V) modes. Under the same incident light intensity (0.02 mW cm^−2^), the photocurrent in PM mode is much higher. From the transient photoresponse in the PV mode, the rise time of 40 µs and fall time of 550 µs are estimated, as shown in Figure 3g, indicating a superior response speed compared to the PM mode (Figure S6, Supporting Information), which has response times in the millisecond scale due to slow charge trapping and de‐trapping processes. In the PM mode, the isolated acceptor domains in the active layer serve as electron traps that capture the photogenerated electrons, causing the band‐bending effect for hole injection, as illustrated in Figure 2a. The release of trapped electrons is slow thus leading to a slow photocurrent response.

In contrast, the charge photogeneration in the PV mode occurs in the PV active layer, with many fewer traps. The PV active layer is a normal bulk heterojunction with balanced donor/acceptor materials. The photogenerated charges can be separated rapidly under the internal built‐in potential between the donor/acceptor interface and the external bias. The charge photogeneration in the PM active layer is less due to the much less donor/acceptor interface (donor/acceptor weight ratio is 100:3). The PM active layer serves as a hole‐transporting layer, as illustrated in Figure 2b. The PM mode developed in this work relies on photoconductive gain enabled by the external charge injection. It inherently has a slow photoresponse because of the trade‐off between gain and response speed. Theoretically, the longer the electrons are trapped in the acceptor domains, the higher the gain is. The 3 dB cut‐off frequency, defined as the frequency at which the device output attenuates to 0.707 of its output at steady state, is measured and presented in Figure 3h. The 3 dB bandwidth of the PV diode reaches 10 kHz, while that of the PM diode is only 300 Hz.

The total noise current of the detector in PM and PV modes is measured with a spectrum analyzer. The spectral noise in PV mode is lower than that in the PM mode. The total noise level in PV (−0.3 V) and PM (20 V) is 3.6 × 10^−13^ $A/\sqrt{Hz}$ and 4.5 × 10^−10^ $A/\sqrt{Hz}$ at 115 Hz, respectively (Figure 3i). The noise estimated here includes shot noise, thermal noise, and other types of noise. A simple evaluation of the total noise solely based on the shot noise (dark current) may lead to an underestimation of the actual noise level, thus resulting in an overestimation of the detection capability of the detector.^[^ ^33^ ^]^

The specific detectivity (D*) results of the detector operating in PM and PV modes are presented in Figure 3j. D* is a metric that quantifies the detector's sensitivity to weak light signals, which can be calculated by $D^{*}=R\sqrt{A}/S_{n}$, where *R* is the responsivity, *A* is the active area, and *S_n_* is the noise spectral density sampled at the same frequency as *R*.^[^ ^34^ ^]^ It can be observed that the D* data of the detector in PV mode is higher. Despite the higher response in the PM mode, the impact of the higher noise in this mode is more significant, leading to a lower D*. As a result, the D* results in the PV and PM modes are 2.5 × 10^11^ Jones and 1.59 × 10^9^ Jones at 800 nm, respectively. Overall, the detector with both PM and PV dual modes can achieve functional complementarity, making it suitable for various scenarios and enhancing functional integration.

In addition to the successful development of the dual‐mode detector for NIR wavelengths, a visible dual‐mode detector employing two P3HT: PC_71_BM BHJs is developed. An identical back‐to‐back device architecture is employed to test the universality of the design concept developed in this work. The weight ratio for P3HT and PC_71_BM is set at 100:1 and 1:1 for the PM and PV BHJs. The visible dual‐mode detector can operate under positive and negative biases, corresponding to the PM and PV modes. The performances of this visible dual‐mode detector, such as EQE (Figure S7, Supporting Information), current‐voltage characteristics (Figure S8, Supporting Information), transient photoresponse (Figure S9, Supporting Information), and dynamic range (Figure S10, Supporting Information) are investigated. The photoresponse characteristics are similar to those in the PBDB‐T: IEICO‐4F‐based NIR dual‐mode detectors. It is worth noting that the integration of the two operational modes can also effectively expand the dynamic range of the detector (≈140 dB). These results indicate that the design scheme adopted here can be employed in different material systems, providing a solid technical foundation for future multifunctional, high‐performance optoelectronic detections.

### 2.3 Application Demonstrations of the Dual‐Mode Detector

**Figure 4:**
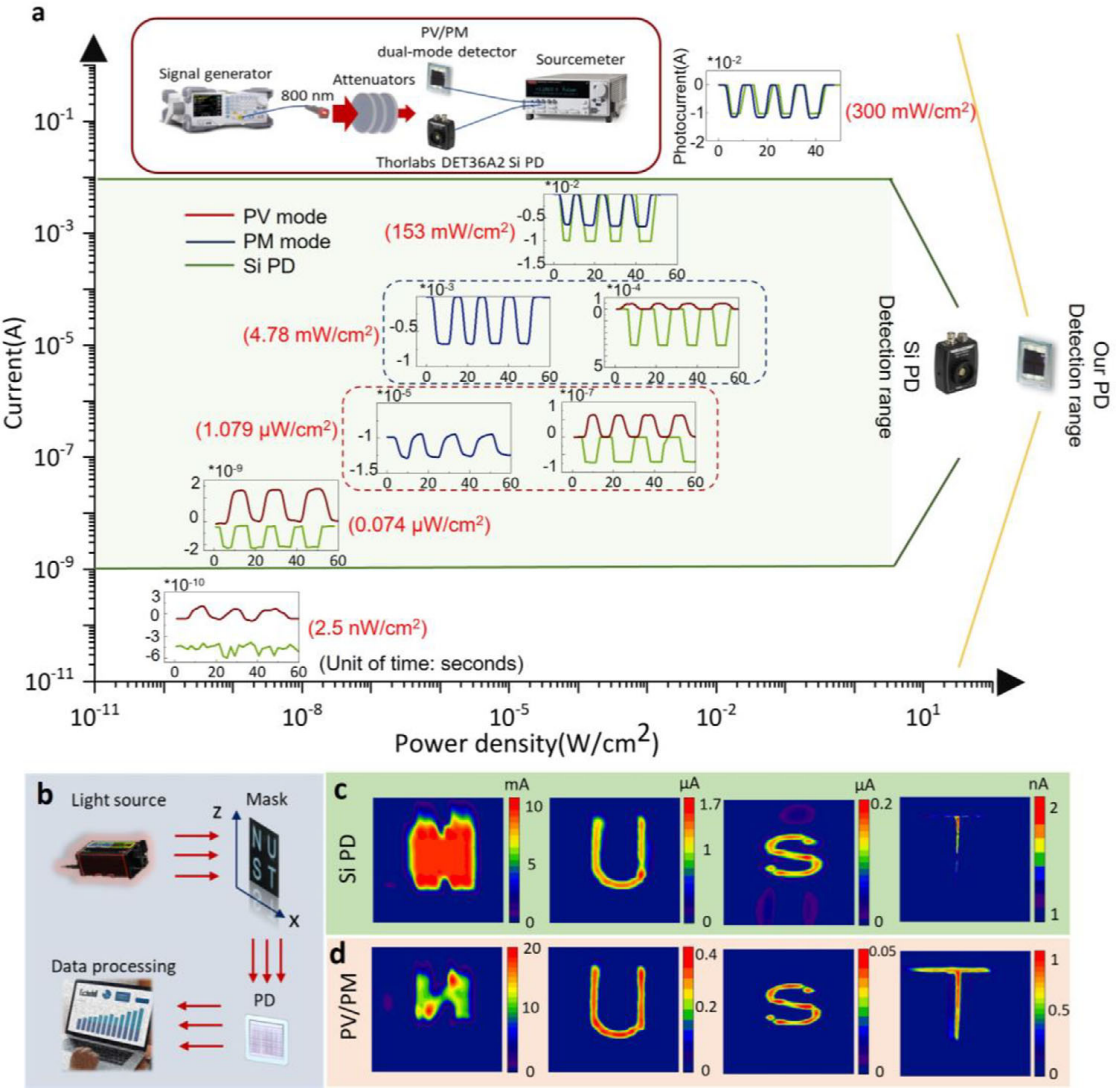
Large dynamic range test of the dual‐mode detector. a) Comparison of transient responses of the dual‐mode detector and a commercial silicon detector (Thorlabs DET36A2) at different NIR light (800 nm) intensities. The green curves represent the photocurrent from the silicon detector, and the red and blue curves represent the photocurrent measured in the PV and PM modes. The inset schematics illustrate the measurement setup. b) Schematics showing the imaging process used here. The 800 nm light source shines through a specially designed mask before reaching the single‐point detector. The single‐point detector records the photocurrent when the mask scans in the X‐Z plane, enabling the image reconstruction of the target mask. The imaging results by using c) the commercial silicon detector, and d) the PM/PV dual‐mode detector. The light transmitted through the four letters exhibits varying intensities: “N” at 500 mW cm^−^^2^, “U” at 0.026 mW cm^−^^2^, “S” at 0.003 mW cm^−^^2^, and “T” at 37 nW cm^−^^2^.

Although the two operating modes exhibit different performances in terms of photoresponse and noise, integrating both modes into a single device can still greatly enhance the adaptability of the detector, especially in terms of dynamic range testing. To verify the large dynamic range characteristics of the dual‐mode detector, its photoresponse capability is compared to a commercial silicon photodetector (Thorlabs DET36A2), as depicted in **Figure** **4**a. Here, the transient photocurrent under modulated NIR (800 nm) light with different intensities is compared. The dual‐mode detector developed in this work has an active area of 0.09 cm^2^, and the active area of the silicon detector is 0.13 cm^2^. The NIR light intensity varies from 2.5 nW cm^−^^2^ to 300 mW cm^−^^2^ achieved by using different attenuators. It can be observed that both types of detectors generate a larger photocurrent as the light intensity increases. However, at extremely low and high light intensities, the two detectors begin to perform differently. It is noticeable that at a sufficiently low incident light intensity of <2.5 nW cm^−^^2^, the silicon detector fails to discern the signal, as photocurrents below 1 nA fall below its detection threshold. In contrast, the dual‐mode detector can leverage the lower noise of its PV mode (bias = −0.3 V) to achieve a noticeable photocurrent response. When the light intensity is sufficiently high, the Si detector begins to exhibit saturation effects in photocurrent. It can be observed that when the light intensity reaches 153 mW cm^−2^, the photocurrent of the silicon detector ceases to increase, remaining essentially consistent with the photocurrent under an intensity of 300 mW cm^−2^. As the light intensity increases, the photocurrent of PV mode gradually saturates. The detector switches to PM mode (bias = 20 V). Although the photocurrent in the PM mode is lower than that of the silicon detector at an intensity of 153 mW cm^−^^2^, it continues to rise as the light intensity increases. Notably, at an intensity of 300 mW cm^−^^2^, the photocurrent of the PM mode becomes comparable to that of the silicon detector. It can be observed that the saturation photocurrent of the PM‐mode detector is greater than that of the silicon detector, not to mention that its active area is even smaller than that of the silicon detector.

As a result, the dynamic range performance of the dual‐mode detector surpasses that of the commercial silicon detector, taking advantage of the two detection modes, which potentially bring revolutionary breakthroughs for infrared detection in extreme conditions. The dual‐mode detector, boasting an exceptionally large dynamic range, can be aptly applied in scenarios that demand such a capability. The imaging capability of the dual‐mode detector is tested and compared to a commercial silicon detector. The measurement setup is schematically illustrated in Figure 4b. The light signal (800 nm) traverses the mask before being captured by the detector, with the mask undergoing scanning along both the X and Z axes. The mask contains four letters, i.e., “N”, “U”, “S”, and “T”. Throughout the imaging process, the detector quantifies the photocurrent at various points across the mask. The subsequent image is then constructed by meticulously processing this 2D dataset. It should be noted that the light transmitted through the four letters exhibits varying intensities: “N” at 500 mW cm^−^^2^, “U” at 0.026 mW cm^−^^2^, “S” at 0.003 mW cm^−^^2^, and “T” at 37 nW cm^−^^2^. Figure 4c,d demonstrates the imaging results recorded by the silicon detector and dual‐mode detector. It is evident that the image of the letter “N” captured by the silicon detector blurs due to the photocurrent saturation at a high light intensity. The light intensity exceeds the detection limit of the silicon detector, resulting in only 10.7 mA of photocurrent being detected by the silicon detector. This causes significant crosstalk in the entire area, making it difficult to identify the letter “N”. On the other hand, the image of the letter “T” appears somewhat unclear, primarily due to the detector's insufficient sensitivity under conditions of very low light intensity. In comparison, the dual‐mode detector provides crisp and distinct images of the four letters, indicating that the dual‐mode detector possesses outstanding large dynamic range imaging capabilities. It is worth noting that the imaging of the letter “N” is in the PM mode, and the imaging of letters “U”, “S”, and “T” is in the PV mode of the dual‐mode detector.

The dual‐mode detector, featuring low noise in its PV mode, can serve as an effective photoplethysmography (PPG) sensor, utilizing ambient light as the illumination source. The setup and PPG signals are illustrated in Figure S11 (Supporting Information). The Supplementary Video demonstrates the PPG measurements. Unlike conventional PPG detectors that require an active light source for illumination, our PPG detector is capable of clearly capturing heart rate signals under indoor light conditions (425 lx), which could reduce system complexity in future applications. These results suggest that the dual‐mode detector is adept at accommodating the detection and imaging demands across varying light intensities, thereby delivering clearer and more precise outcomes for diagnosis and object identification.

## 3 Conclusion

In summary, the concept and design roles of constructing large dynamic dual‐mode detectors by integrating PM and PV‐type detecting units in a back‐to‐back structure are developed. By capitalizing on the low noise level of the PV mode and the high saturation photocurrent of the PM mode, the dual‐mode detector is capable of detecting an exceedingly broad range of light intensities, spanning from 10^−10^ to 10 W cm^−^^2^. This capability eclipses the performance of a commercial silicon detector, offering a significant advancement in the detection of light intensity across a wide spectrum. Distinguished from traditional detectors, the dual‐mode detector achieves superior performance through bidirectional biasing, enabling straightforward adjustment of the detector's functionalities by simply tweaking the voltage in practical applications, providing flexibility and precision. The successful development of the PV/PM dual‐mode detectors enhances the functionality and performance of solution‐processable semiconductor‐based optoelectronic devices, laying a crucial foundation for further advancements and applications in optoelectronic technology.

## 4 Experimental Section

### Device Fabrication

PBDB‐T, IEICO‐4F, and other organic materials were purchased from Advanced Election Technology CO., Ltd. ITO‐coated glass substrates with a resistance of 15 ohms per square were sequentially washed in an ultrasonic bath with diluted detergent, deionized water, acetone, and isopropanol, each for 10 min.

For the PM active layer, the donor PBDB‐T was dissolved in dichlorobenzene at a concentration of 20 mg mL^−1^ in a mass ratio of 100:3 along with the acceptor material, IEICO‐4F, and placed in a glove box and heated to 100 °C with stirring overnight. For the PV active layer, the donor‐acceptor mixture was dissolved in dichlorobenzene at a concentration of 20 mg mL^−1^ and a mass ratio of 1:1, heated to 100 °C, and stirred overnight. The device structure is ITO/PBDB‐T: IEICO‐4F (100:3, 450 nm)/PEDOT: PSS (100 nm)/PBDB‐T: IEICO‐4F (1:1, 250 nm)/LiF (2 nm)/Al (100 nm), and the PM organic film was first deposited on the surface of ITO in a glove box, rotated at 1000 rpm for 60 s and annealed on a thermal evaporator for 10 min. The PEDOT: PSS layer was prepared by spin‐coating at 1000 rpm for 100 s. It was then annealed on a thermal evaporator for 10 min. The PV organic film was prepared in the glove box by spin‐coating at 1200 rpm and was then annealed for 10 min before transferring to a thermal evaporation chamber for vacuum deposition of the LiF, Al layer. Finally, the films were encapsulated before measurements. The active area of each device is 9 mm^2^.

### Device Characterization

All devices were characterized under ambient conditions. The current‐voltage characteristics of the detector devices were measured under dark and light conditions using a current‐source meter (Keithley 2450). An 800 nm NIR LED was employed as the light source. For light intensity over 30.4 mW cm^−2^, an 800 nm NIR laser is used. A xenon lamp integrated with a monochromator‐generated monochromatic light was used for EQE measurements. A Tektronix MSO 4054B mixed‐signal oscilloscope was used to measure the transient photocurrent of the dual‐mode detector. The modulated transient light was from a RIGOL DG1062 waveform generator‐controlled LED, in the measurement of transient light response. The optical field distribution was simulated using the finite‐difference time‐domain (FDTD) method. The film thickness was measured using a DektakXT step profiler. The measurement of noise current of the detector operating in PM and PV modes was conducted in a darkroom environment. The device is biased by a sourcemeter, and its noise is measured by a spectrum analyzer (Tektronix RSA5106A).

## Conflict of Interest

The authors declare no conflict of interest.

## Supporting information



Supporting Information

## Data Availability

The data that support the findings of this study are available from the corresponding author upon reasonable request.
