# Supplementary material for: Photovoltaic and Photomultiplication Dual‐Mode Near‐Infrared Organic Detectors with Large Dynamic Range for Intensive and Faint Light Sensing
Source: Adv Sci (Weinh). 2025 May 30;12(32):e06499. doi: 10.1002/advs.202506499 (PMC12407299; doi:10.1002/advs.202506499)
Supplement: Supplementary file 1 — Supporting Information [file ADVS-12-e06499-s001.docx]

**Supporting Information**

**Photovoltaic and Photomultiplication Dual-mode Near-infrared Organic Detectors with Large Dynamic Range for Intensive and Faint Light Sensing**

Xin Hu^‡^, Ning Li^‡,^*, Mingyang Ren, Yifan Ji, Hongmei Guo, Qian Chen, Xiubao Sui*

X. Hu, N. Li, M. Ren, Y. Ji, Q. Chen, X. Sui

School of Electronic and Optical Engineering, Nanjing University of Science and Technology, 200 Xiaolingwei Street, Nanjing, 210094, China.

E-mail: liningbox@outlook.com, sxb@njust.edu.cn

H. Guo

School of Computer and Electronic Information, Nanjing Normal University, Nanjing 210023, China.

^‡^ These authors contributed equally to this work.

**Supporting Figures:**


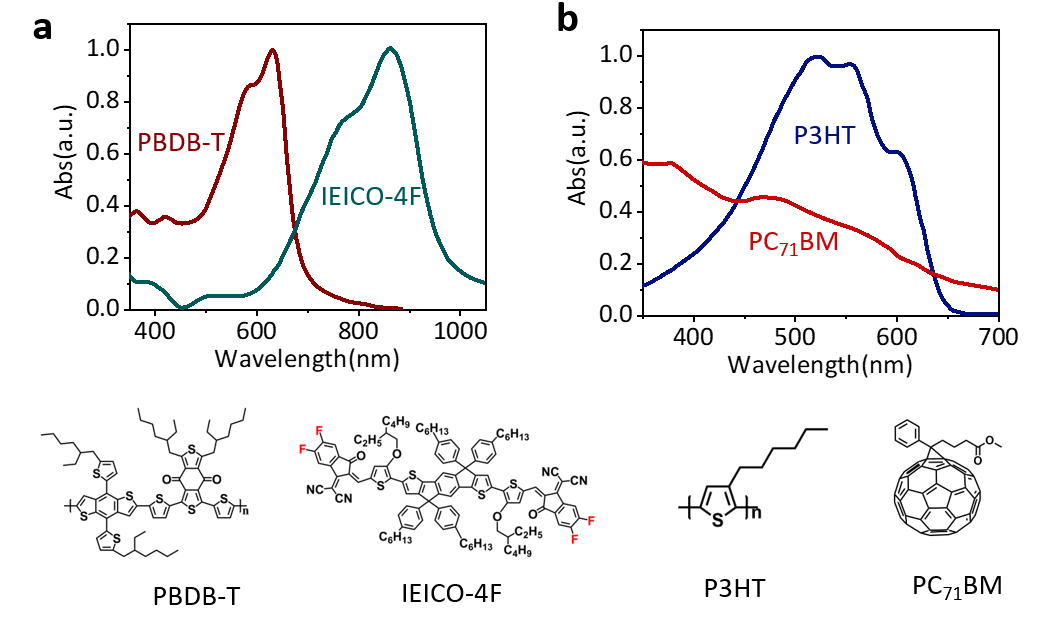


***Figure S1.*** *Absorption of the materials (PBDB-T, IEICO-4F, P3HT, and PC_71_BM) used in the active layer for (a) the IR detector and (b) the visible detector. The molecular structures of the four materials.*


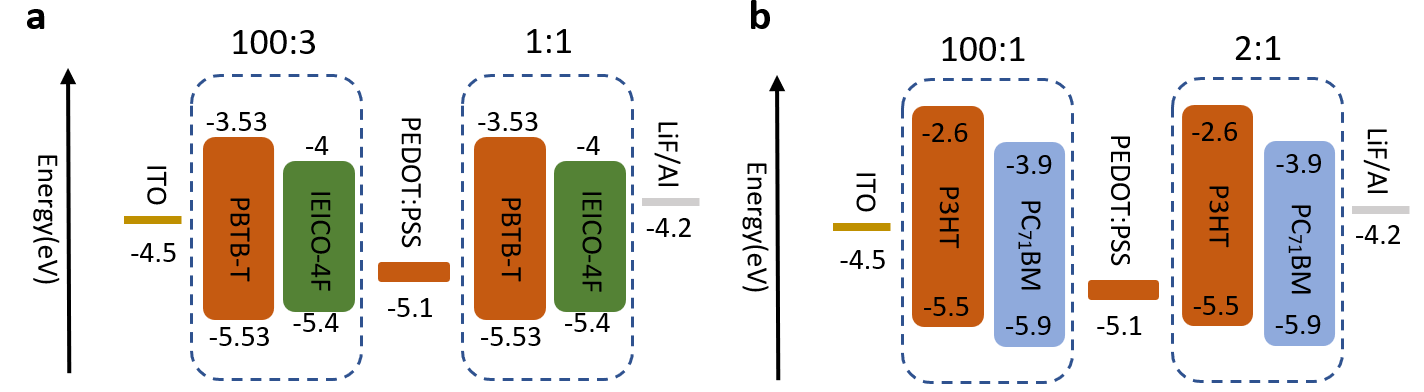


***Figure S2.*** *The energy level diagrams for the materials in (a) the IR detector (PBDB-T and IEICO-4F are used as the BHJ) and (b) the visible detector (P3HT and PC_71_BM are used as the BHJ).*


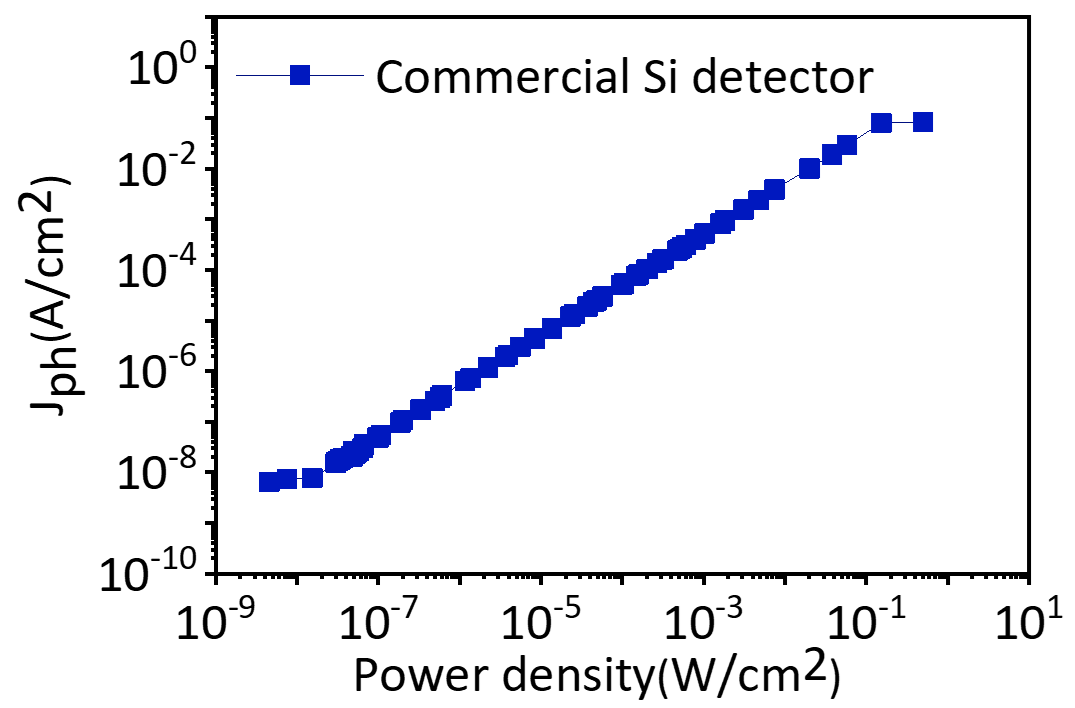


***Figure S3.*** *The dynamic range of a commercial silicon detector.*


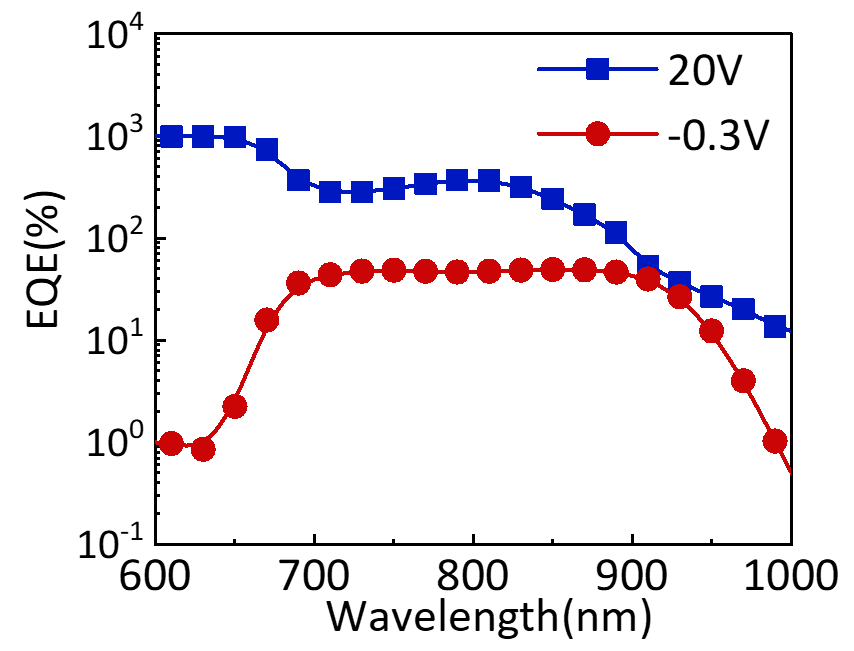


***Figure S4.*** *The comparison of EQE results between the PV mode and PM mode of the dual-mode detector.*

**
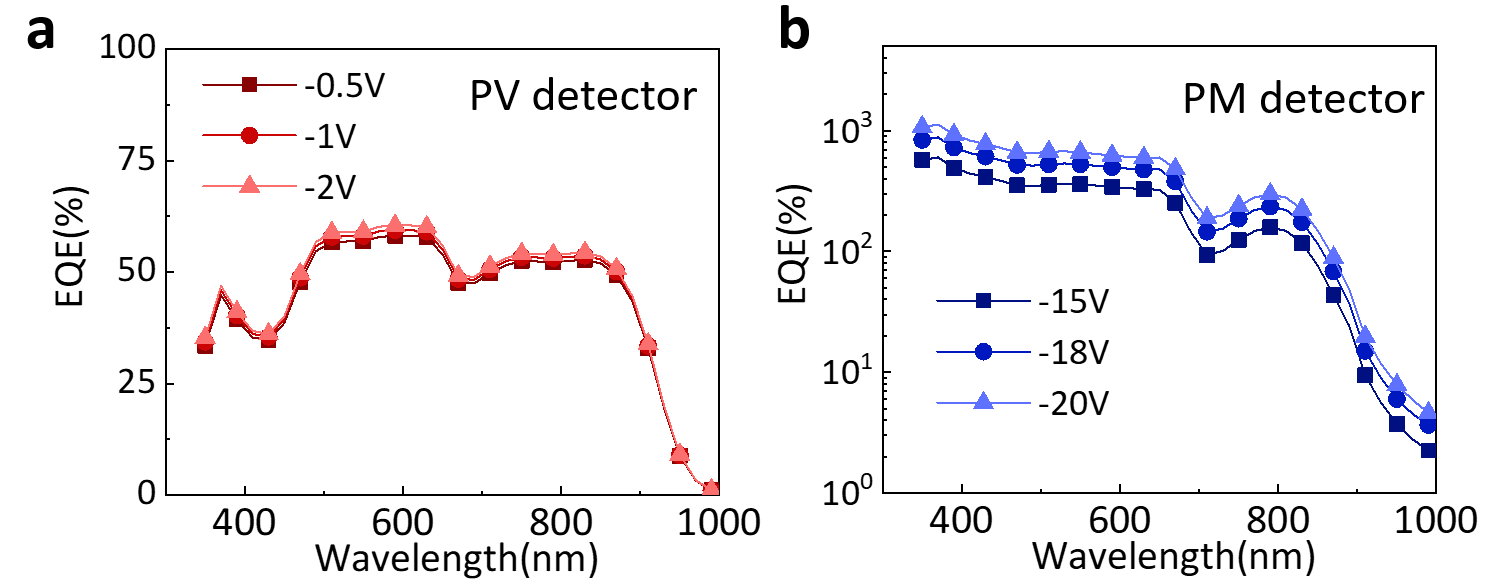
**

***Figure S5.*** *The EQE results of the two single-mode control detectors, (a) for the PV detector and (b) for the PM detector, as a function of wavelength. The device structure for the PV detector is ITO/ PEDOT: PSS/ PBDB-T: IEICO-4F (1:1)/LiF/Al.* *The device structure for the PM detector is ITO/PBDB-T: IEICO-4F (100:3)/ PEDOT: PSS/LiF/Al.*


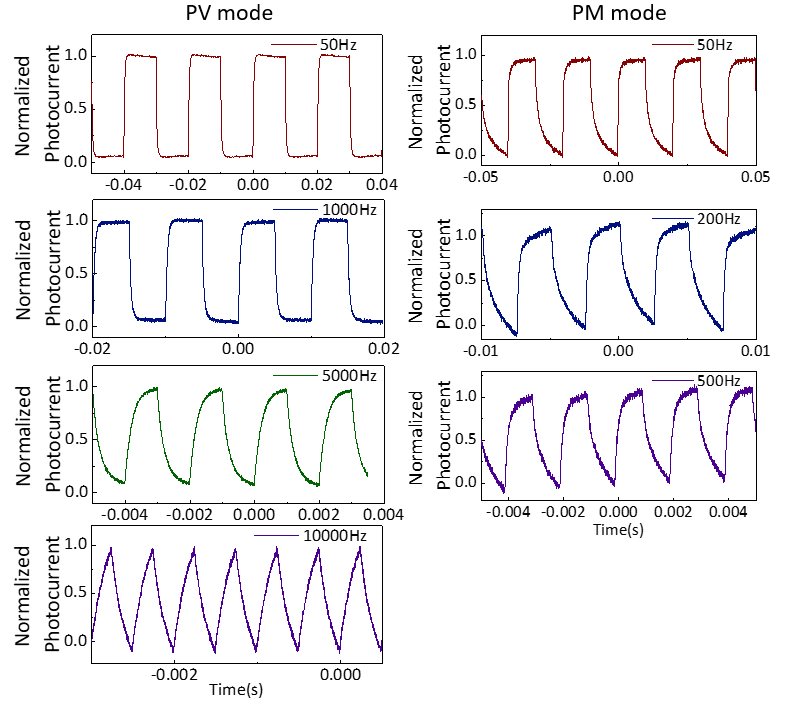


***Figure S6.*** *The transient photoresponse of the two operational modes of the dual-mode detector. Left: Photocurrent response in the PV mode. Right: Photocurrent response in the PM mode.*


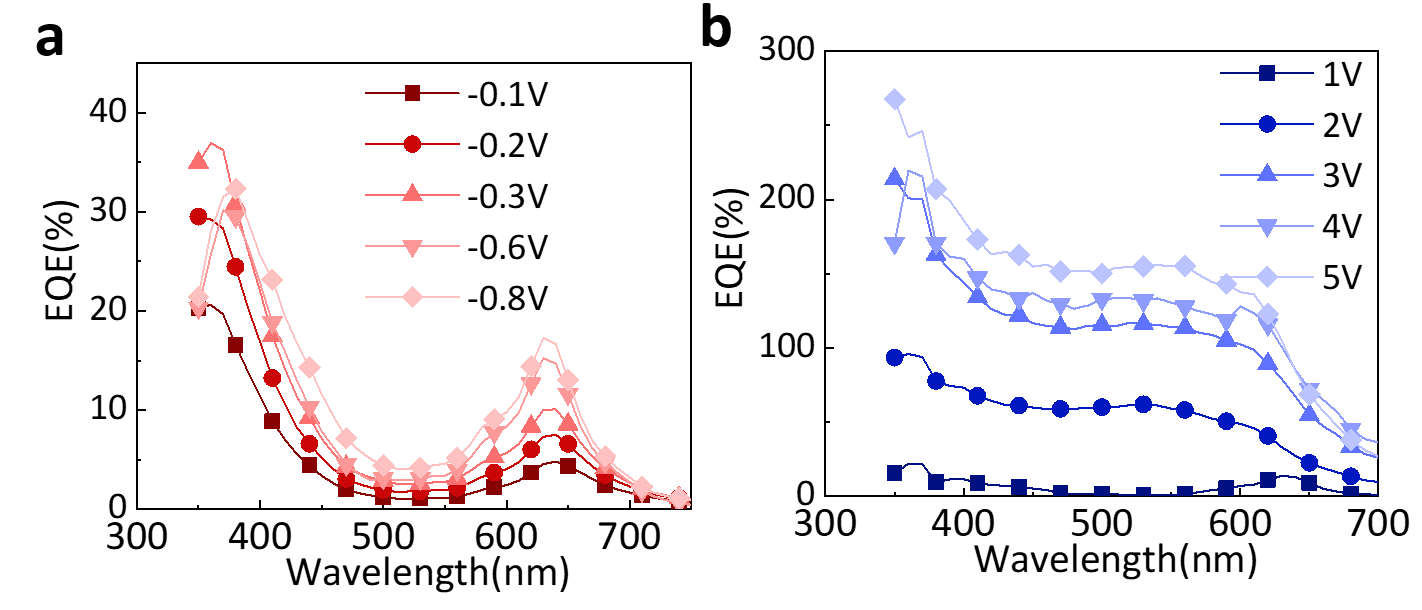


***Figure S7.*** *The EQE results of (a) the PV mode and (b) the PM mode in the dual-mode visible detector based on P3HT:PC_71_BM BHJ, as a function of bias.*


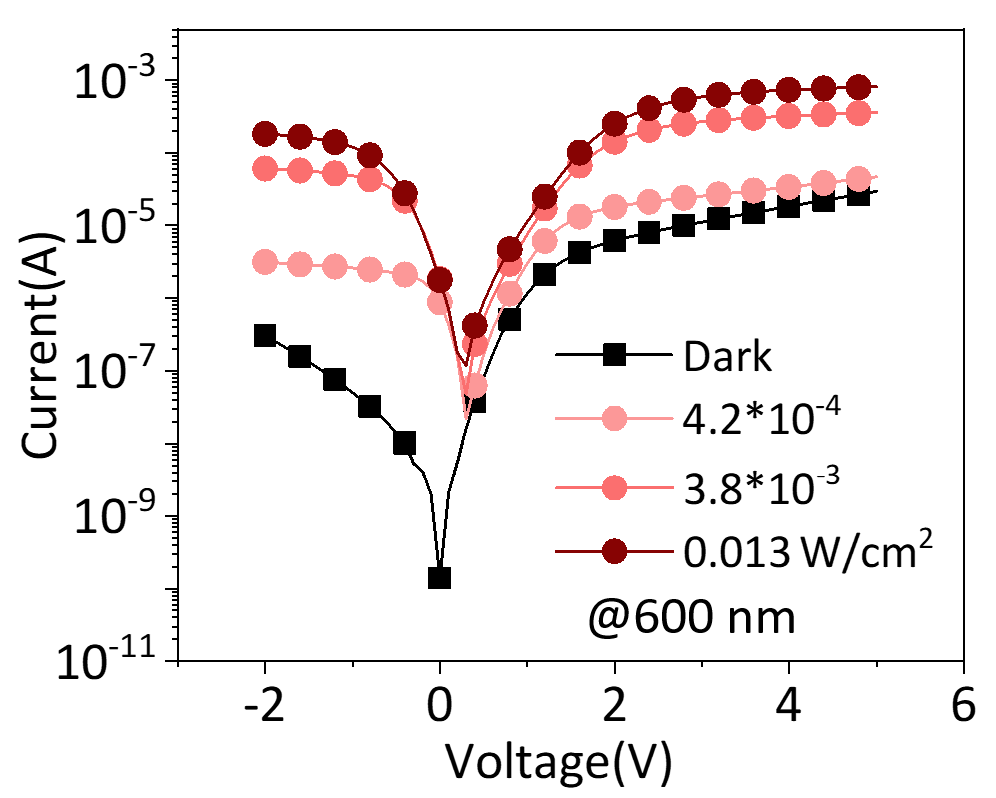


***Figure S8.*** *The current-voltage curves of the dual-mode visible detector under different light intensities.*


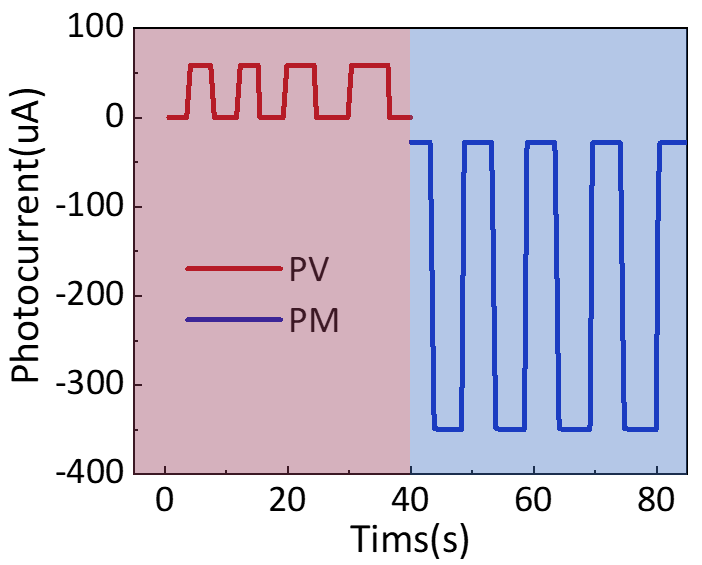


***Figure S9.*** *The transient photocurrent of the dual-mode visible detector measured under 600 nm light in PV and PM modes.*


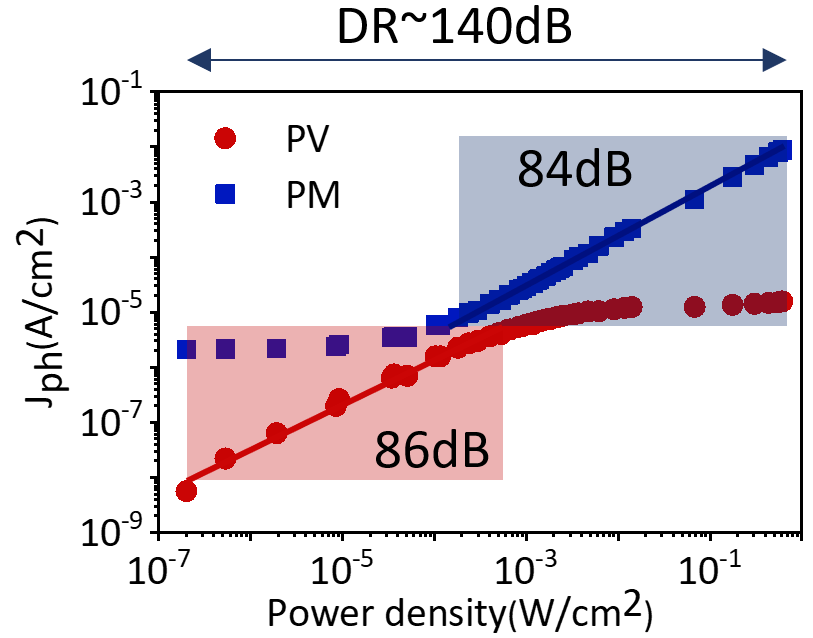


***Figure S10.*** *The dynamic range of the dual-mode visible detector.*

*
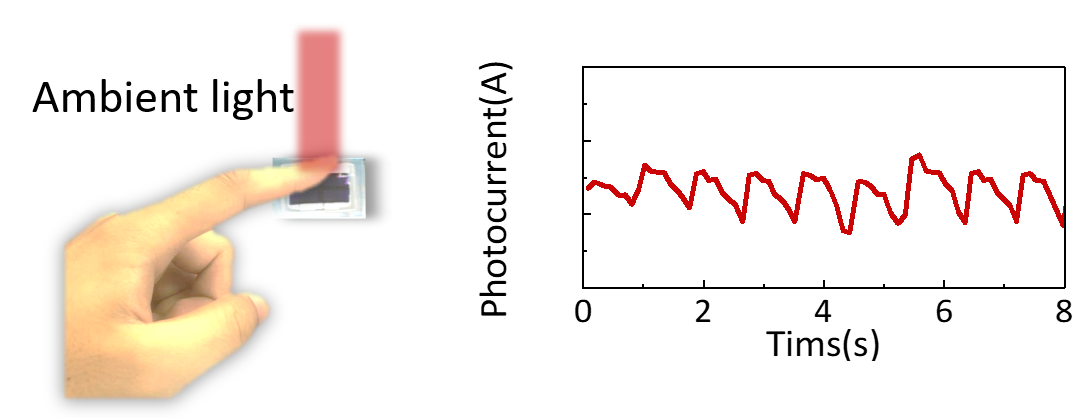
*

***Figure S11.*** *PPG measurement setup and results under indoor low light conditions.*
